# Quantification of serum elastase inhibitory activity in patients with pulmonary emphysema with and without alpha-1 antitrypsin deficiency

**DOI:** 10.1371/journal.pone.0324237

**Published:** 2025-06-05

**Authors:** Gerard Orriols, Cristina Aljama, Francisco Rodriguez-Frias, Pablo-Gabriel Medina, Roser Ferrer-Costa, Galo Granados, Alexa Nuñez, Ane López-González, Gerardo Ruiz-Satelinas, Marc Miravitlles, Miriam Barrecheguren, Cristina Esquinas

**Affiliations:** 1 Department of Pneumology, Hospital Universitari Vall d’Hebron/ Vall d’Hebron Research Institute (VHIR), Vall d’Hebron Barcelona Hospital Campus, Barcelona, Spain; 2 Department of Medicine, Universitat Autònoma de Barcelona, Barcelona, Spain; 3 Department of Basic Sciences, Universitat Internacional de Catalunya, Barcelona, Spain; 4 Clinical Biochemistry Research Group, Vall d’Hebron Research Institute (VHIR), Vall d’Hebron Barcelona Hospital Campus, Barcelona, Spain; 5 Liver Unit, Hospital Universitari Vall d´Hebron, Vall d’Hebron Barcelona Hospital Campus, Barcelona, Spain; 6 Department of Clinical Biochemistry, Hospital Universitari Vall d’Hebron, Vall d’Hebron Barcelona Hospital Campus, Barcelona, Spain; 7 Department of Public Health, Mental Health and Maternal and Child Health Nursing, Faculty of Nursing, University of Barcelona (UB), Barcelona, Spain; Medizinische Fakultat der RWTH Aachen, GERMANY

## Abstract

Pulmonary emphysema, a component of chronic obstructive pulmonary disease (COPD) is characterised by irreversible alveolar tissue destruction and is produced by an imbalance between proteolytic enzymes, mostly neutrophil elastase (NE), and its inhibitors, mainly alpha-1 antitrypsin (AAT). We measured elastase-inhibitory activity (EIA) in serum samples and determined whether there is an association between EIA and COPD severity. This cross-sectional study recruited COPD patients with and without severe alpha-1 antitrypsin deficiency (AATD) and healthy controls. A semi-automated method assessed EIA using a porcine elastase inhibition assay. EIA levels and the EIA/AAT ratio were compared across groups and the correlation with clinical variables was analysed. A total of 86 individuals were recruited: 36 COPD patients, 20 individuals with COPD associated with AATD (Pi*ZZ mutation), of whom 11 were on augmentation therapy, and 30 healthy controls. Positive, linear and significant relationships were observed between EIA and AAT levels. The EIA/AAT ratio was higher in non AATD-related COPD patients compared to untreated Pi*ZZ patients and controls. Further analysis in non-AATD-related COPD patients revealed a higher EIA/AAT ratio associated with older age, higher comorbidity burden and a trend towards higher severity of lung disease. The strong correlation between AAT levels and EIA suggests that the assay technique employed is robust and effective for assessing EIA. The EIA assay may serve as a potential biomarker for the assessment of the severity and prognosis of emphysema.

## Introduction

Chronic obstructive pulmonary disease (COPD) is characterised by persistent respiratory symptoms, chronic inflammation and tissue destruction, by means of various and complex pathophysiological mechanisms [1]. Among these, pulmonary emphysema, a critical component of COPD, involves the irreversible destruction of alveolar tissue, leading to decreased airflow and impaired gas exchange [2,3]. The pathogenesis of emphysema is closely related to an imbalance between proteolytic enzymes (proteases) and their physiological inhibitors (anti-proteases) [4].

Serine proteases are the most important group of proteolytic enzymes in the lungs and are expressed and released by activated neutrophils during inflammatory response [5]. Among these, neutrophil elastase (NE) is one of the most abundant serine proteases [6]. It plays a crucial role in microbial killing and modulating immunological response [7]. NE degrades elastin, a key structural extracellular matrix (ECM) protein that is responsible for the elasticity and structural integrity of lung tissue, contributing to ECM remodelling and tissue repair. NE can also increase proinflammatory cytokines, such as Interleukin-8, by the activation of the Toll-like receptor-4 or epidermal growth factor cell signalling pathways [8].

Under normal physiological conditions, elastase-inhibitory activity (EIA) is tightly modulated by anti-elastases, such as alpha-1 antitrypsin (AAT), which mainly inhibits NE and prevents excessive elastin degradation [9]. The regulation of NE is important for maintaining lung tissue integrity and function. However, in pulmonary diseases, this delicate balance may be disrupted [10]. Factors contributing to this imbalance include genetic deficiencies (e.g., alpha-1 antitrypsin deficiency -AATD-), chronic inflammation, and environmental exposures, such as smoking, leading to insufficient inhibition of NE. This elastase/anti-elastase imbalance leads to increased degradation of lung elastin and the subsequent development of emphysema [11,12].

The most important genetic factor related to the development of emphysema is AATD [13,14], which is caused by mutations in the *SERPINA1* gene, resulting in reduced serum and tissue AAT levels, and therefore, insufficient inhibition of NE. The most common and physiologically normal allele is the M allele, while Z (Glu342Lys) and S (Glu264Val) mutations are the most common deficient variants [15].

Alpha-1 homozygous Pi*ZZ (for proteinase inhibitor) individuals have low circulating levels of AAT (by 10–15% lower compared to normal) and have an increased risk of emphysema [16]. The only specific treatment available for emphysema associated with AATD is the intravenous infusion of AAT purified from human plasma donors; i.e., augmentation therapy, which raises serum AAT levels above the theoretically protective threshold level of 11 μM/L, delaying the progression of emphysema [13,14].

However, not all AATD patients develop emphysema of the same degree of severity and, on the other hand, individuals with normal AAT levels may develop early and severe emphysema. EIA may be an important modulating factor, since EIA may also be reduced even in the presence of normal or elevated levels of AAT but with an altered functionality; for example, when the methionine of the reactive site is oxidized and, consequently, not recognized by the NE [17]. Therefore, measurement of EIA may provide important prognostic information for lung disease. Given the critical role of NE in the pathogenesis of COPD and AATD-related emphysema, accurate measurement of serum EIA is crucial.

We performed a semi-automated method for measuring EIA in serum samples and determined whether there is an association between EIA and the severity of lung disease in patients with COPD with and without AATD.

## Methods

### Design

This was a cross-sectional study conducted in patients attending the outpatient clinic of the Pneumology Department at Vall d’Hebron University Hospital (Barcelona, Spain) from November 2020 to May 2022.

### Participants

Patients were consecutively recruited according to the following four groups: 1) patients with a previous diagnosis of COPD and Pi*MM or Pi*MS genotype (both considered normal and not related to severe AATD); 2) patients with COPD and severe AATD (Pi*ZZ genotype) not on augmentation therapy; 3) patients with COPD and severe AATD (Pi*ZZ) on augmentation therapy with plasma purified AAT (Prolastin® or Trypsone®; Grifols, Barcelona, Spain) at a dose of 120 mg/kg every 15 days; 4) healthy individuals as a control group recruited during the same period with the following criteria: adults older than 40 years, never smokers with no history of lung disease and a Pi*MM or Pi*MS genotype.

The inclusion criteria for the COPD group were: patients over 40 years of age, smokers or ex-smokers with a cumulative smoking consumption of at least 10 pack-years and a post-bronchodilator forced expiratory volume in 1 second/forced vital capacity (FEV1/FVC) ratio <0.7 [18]. All the patients presented clinical stability at the time of recruitment in the study, defined as no respiratory exacerbations in the previous 2 months.

All individuals had undergone genotyping by sequencing of all coding exons of *SERPINA1* [19]. Individuals with rare variants or intermediate deficiencies (Pi*SS, Pi*SZ, Pi*MZ) were excluded from this study.

The study was approved by the Ethics Committee of the Vall d’Hebron Hospital (Barcelona, Spain, number PR (AG) 102/2019) and all participants provided written informed consent. All the data were confidential and privacy was preserved by assigning each participant a code for the statistical processing of the data, as well as adhering to the principles set out in EU Regulation 2016/679 of the European Parliament and of the Council of April 27, 2016 on the protection of personal data and the free movement of data “and Spain’s Organic Law” Organic Law 3/2018 of 5 of December for the Protection of Personal Data and the Guarantee of Digital Rights. The study was conducted in accordance with the Declaration of Helsinki.

### Variables

Sociodemographic and clinical data were collected from all the patients. Comorbidities were registered according to the Charlson comorbidity index (CCI) [20]. Lung function tests were performed according to standardized recommendations [21]. Respiratory symptoms were assessed using the degree of dyspnoea according to the modified Medical Research Council (mMRC) dyspnoea scale [22] and the COPD Assessment Test (CAT) score [23]. In addition, the impact of the disease was calculated with the BODEx (body mass index, obstruction, dyspnoea and exacerbations) index [24]. The following biomarkers were analysed: C-reactive protein (CRP), AAT and fibrinogen.

### Elastase inhibitory activity (EIA)

Peripheral blood samples were collected from all participants and stored at -80^o^C until analysis. Blood samples were taken immediately before the next infusion in patients receiving augmentation therapy to avoid the interference of exogenous M protein in the results as much as possible [25].

Plasma was isolated by centrifugation at 3,500 x g for 10 minutes at room temperature. First, total AAT protein in plasma samples was measured by immunonephelometry on a BNII instrument (Siemens Healthcare, Malvern, PA, USA). Plasma samples were assayed by the porcine elastase inhibitory assay [26]. In this method, EIA is determined using a kinetic spectrophotometric method based on the principle of inhibition of hydrolytic activity of porcine pancreatic elastase on a chromogenic substrate.

Briefly, plasma samples were diluted with tris buffer with human serum albumin and 100 µl were added to a microplate. Reaction was initiated by adding 50 µl of porcine pancreatic elastase in excess to individual wells, followed by incubation at room temperature for 10 min. During that time, the AAT contained in the samples formed an irreversible complex with part of the elastase. Finally, 50 µl of a specific chromogenic substrate (N-succinyl Ala-Ala-Ala p-nitroanilide, Sigma Aldrich) was added and incubated for 3 minutes. The remaining uncomplexed elastase hydrolyses the elastase substrate into a yellow compound absorbed at 405 nm. The reaction was stopped with acetic acid 50% and the plate was read after 2 min. From the absorbance readings, the concentration of functionally active AAT was determined by interpolation from a calibration curve constructed with an AAT standard. Buffer used for sample dilution was added instead of a sample as a negative control (no elastase inhibition, maximum absorbance), and a pool of serum sample with a known EIA was used as a positive control (elastase inhibition, low absorbance).

The standards for functionally active AAT and the pool of serum samples with a known EIA were provided by Grifols (Barcelona, Spain). The technique used in this essay is suitable for open analysers and thus, it was adapted and carried out in a Triturus ELISA instrument (Grifols. Barcelona, Spain), a completely open and automated ELISA analyser. All samples were analysed in triplicate and EIA was expressed as the concentration of functionally active protein in mg/mL.

### Quality assessment

Two parameters were used as quality assessment for all the experimental plates analysed: A) A sample with known EIA was used as the external control for quality assessment in each experiment. The results obtained from this external control in all the analysed plates are within the optimal range of activity according to the manufacturer’s protocol. Moreover, alarm and rejection limits were established. B) A single internal control, consisting of a pool of serum samples from healthy subjects, was used across all the analysed plates to verify technique deviation.

### Statistical analysis

Qualitative variables were described with absolute frequencies and percentages. The description of quantitative variables was performed using the mean, standard deviation (SD) and median. The Kolmogorov–Smirnov test was used to assess the normality of distributions.

The sociodemographic, clinical characteristics, serum AAT levels (mg/dL) and EIA (mg/mL) were compared among the groups. In the case of quantitative variables, Kruskal-Wallis tests, with Bonferroni correction for multiple comparisons were carried out. The Chi-squared test (Fisher test for frequencies <5) was used for the comparison of categorical variables. Linear relationships between clinical variables and levels of AAT and EIA were also analysed using the Spearman correlation coefficient.

The EIA/AAT ratio indicates the activity per milligram of AAT protein. This specific or relative activity was calculated as the ratio of concentration of EIA to total AAT protein determined by immunonephelometry.

The EIA/AAT ratio was transformed into a binary variable using the non AATD-related COPD group median value as the cut-off (<and>= 1.3) and comparisons between both groups (above and below the median activity) were performed. For all the tests, p-values < 0.05 were considered statistically significant. The statistical package R Studio (V4.3.3) was used for the analyses.

## Results

### Population characteristics

A total of 86 individuals were recruited: 36 with COPD and normal AAT levels, 20 patients with COPD and AATD (Pi*ZZ), of whom 11 were on augmentation therapy, and 30 healthy controls.

The mean age of non AATD-related COPD patients was 71.2 (SD: 10.1) years, 78.9% were men and 3 (8.1%) were active smokers, 32 (88%) were Pi*MM and 4 (12%) were Pi*MS. The mean forced expiratory volume in one second as percent predicted (FEV1%) was 52.3% (SD: 21%) and the mean CCI was 5.3 (SD: 1.8) (Table 1). The control group consisted of 30 never smokers, with a mean age of 53.7 (7.9) years, and 18 (60%) were females; 25 (83%) were Pi*MM and 5 (17%) were Pi*MS. Respiratory diseases and significant comorbidities were excluded.

**Table 1:** Sociodemographic and clinical characteristics according to the study groups.

Variables	COPD (N = 36)	Pi*ZZ-COPD not on augmentation therapy (N = 9)	Pi*ZZ-COPD on augmentation therapy (N = 11)	p value
Age, years	71.5 (10.1)	67.9 (9.6)	62 (11.7)	<0.001^1^
Sex, male (%)	29 (78.4)	3 (33.3)	5 (45.5)	0.005^2^
Former smoker (%) Active smoker (%)	33 (91.7) 3 (8.3)	4 (44.4) 0 (0)	11 (100) 0 (0)	<0.001^2^
Smoking, pack-years	43.5 (19.5)	9.4 (6.3)	22.1 (9.3)	<0.001^1^
Charlson comorbidity index	5.3 (1.8)	3.8 (1.3)	3.1 (1.3)	<0.001^1^
BODEx index	3.19 (2.01)	1.67 (1.12)	4.09 (1.64)	0.013
FEV1 (%)	53.6 (20.8)	65.1 (15)	31.1 (12.9)	<0.001^1^
Patients with exacerbations in the previous year (%)	8 (22.2)	1 (11.1)	3 (27.3)	0.780^2^
BMI (kg/m2)	28.2 (5)	23.7 (3.5)	24.0 (2.3)	0.008^1^
CAT score	13.5 (5.7)	12 (8.8)	17 (5.8)	0.013^1^
MRC dyspnoea score	2.2 (1.1)	1.3 (0.7)	2.1 (0.9)	0.052^1^
Treatment with ICS (%)	18 (50%)	3 (33.3)	8 (72.7)	0.201^2^
Treatment with LABA (%)	33 (91.7%)	6 (66.7)	10 (90.9)	0.119^2^
Treatment with LAMA (%)	33 (91.7%)	6 (66.7)	10 (90.9)	0.119^2^
Fibrinogen (mg/dL)	430 (73.7)	407.7 (51.3)	383.4 (67.8)	0.316^1^
Leukocytes (x10^9/L)	7.8 (2.3)	7.4 (2.8)	7.2 (1.6)	0.889^1^
Haemoglobin (g/dL)	14 (2.5)	14.4 (1.9)	15.3 (1.3)	0.331^1^
C-reactive protein (mg/L)	0.97 (1.4)	0.33 (0.2)	0.26 (0.2)	0.010^1^

Footnote: Values are mean (standard deviation) unless otherwise specified. BODEx: Body mass index, airflow Obstruction, Dyspnoea and Exacerbations; FEV1: Forced expiratory volume in 1 second; BMI: Body Mass Index; CAT: COPD Assessment Test; MRC: Modified Medical Research Council; ICS: inhaled corticosteroids; LAMA: long-acting antimuscarinic agents; LABA: long-acting beta-2 agonists.

^1^p-values obtained from the Kruskal-Wallis test. ^2^ P-values obtained from the Chi-square test or the Fisher’s exact test.

Patients with AATD-related COPD were younger, more frequently female and with a lower smoking exposure compared to non AATD-related COPD patients. In particular, patients on augmentation therapy had the most impaired FEV1(%) and a worse BODEx. Regarding biomarkers, AATD patients presented lower CRP levels (Table 1).

### Antielastase activity and correlation with AAT levels in all groups

The EIA was found to be within the established lower and upper limits in all the controls analysed. Moreover, the coefficient of variation calculated with this internal control across all experiments was 4.4%.

**Fig 1:**
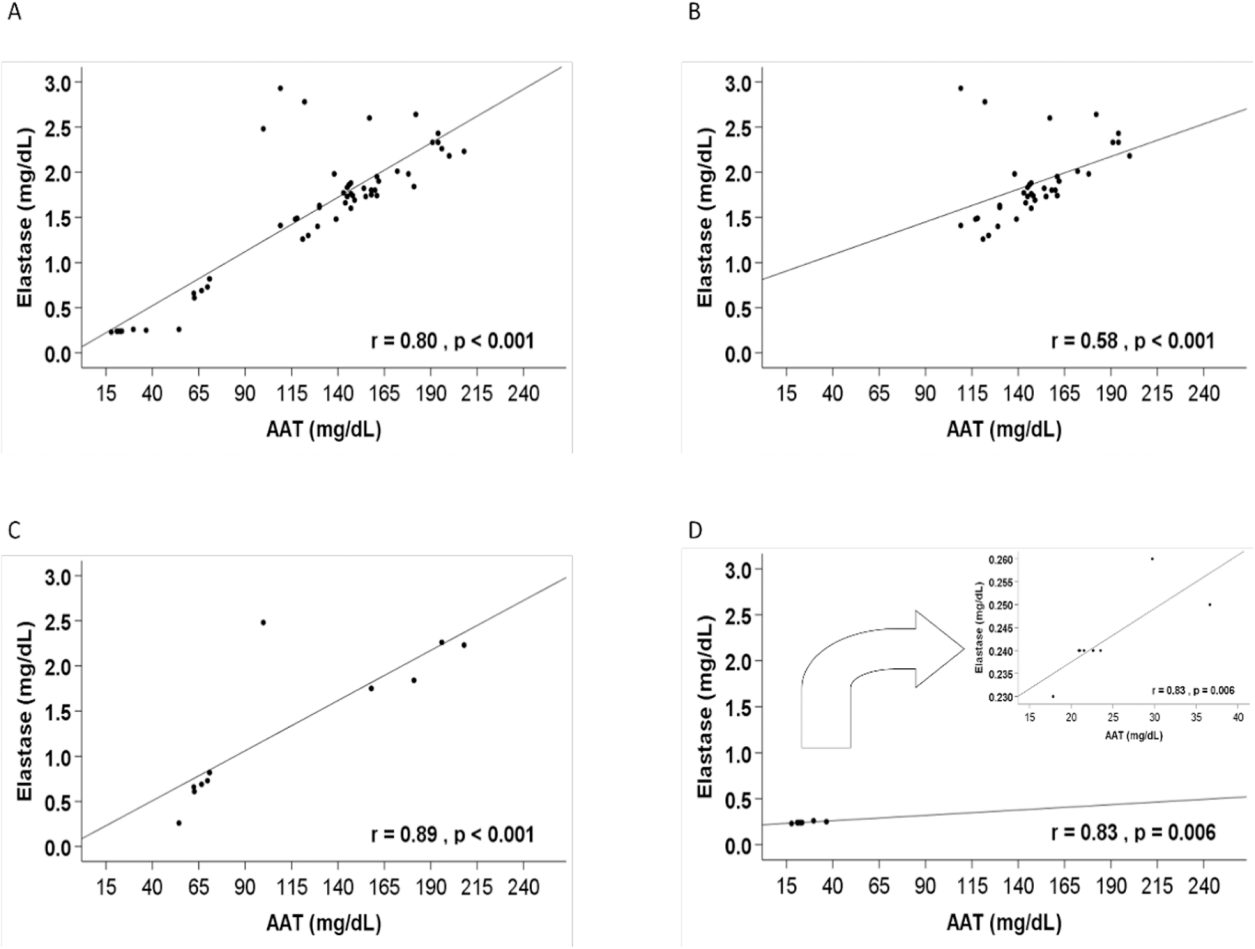
Linear relationship between elastase inhibitory activity and AAT levels. Footnote: A) All patients (not included the control group) (N = 56); B) Non AATD-related COPD (N = 36); C) Pi*ZZ- COPD with augmentation therapy (N = 11); D) Pi*ZZ-COPD without augmentation therapy (N = 9).

A positive, linear and significant relationship between AAT levels and EIA was observed in all groups (r = 0.83, p = 0.006 for untreated Pi*ZZ; r = 0.89, p < 0.001 for treated Pi*ZZ and r = 0.58, p < 0.001 for non-AATD related COPD patients) (Fig 1). However, two patients from the non AATD-related COPD group showed discordant values, with the highest EIA values but relatively lower serum AAT levels. These two cases had the following characteristics: 1) The first presented 2.93 mg/mL of absolute EIA and 109 mg/dL of serum AAT concentration. He was an 86-year-old male former smoker of 25 pack-years and a Pi*MS genotype. He presented several comorbidities: hypertension, dyslipidaemia, tracheobronchomalacia, anxiety and depression. 2) The second presented 2.78 mg/mL of absolute EIA and 122 mg/dL of AAT. He was a 65-year-old male active smoker with a Pi*MS genotype. He had a relevant oncologic history with urothelial carcinoma and non-small cell lung carcinoma and also presented several comorbidities: hypertension, dyslipidaemia, ischaemic cardiopathy and sleep apnoea syndrome.

### Comparison of alpha1 antitrypsin (AAT) serum levels, elastase inhibitory activity (EIA) and EIA/AAT ratios among groups

**Fig 2:**
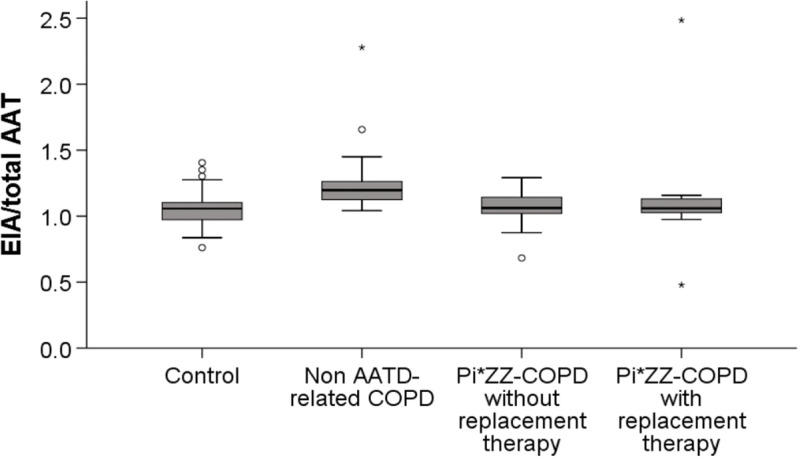
Elastase inhibitory activity (EIA) and Alpha1 antitrypsin (AAT) ratios among groups. Footnote: In each In each box plot, the median value is indicated by the centre horizontal line, and the 25th and 75th percentiles are indicated by the lower and upper box horizontal lines. Whiskers above and below the box indicate the 90th and 10th percentiles. Extreme and mild outliers are marked with an asterisk (*) or a circle (O) on the box plot, respectively. Kruskal-Wallis test indicated a significant difference among the four cohorts (P<0.001). Bonferroni test showed adjusted P<0.05 for comparison between; 1) Control and non AATD-related COPD, 2) Non AATD-related COPD and Pi*ZZ-COPD not on augmentation therapy, and 3) Non AATD-related COPD and the Pi*ZZ-COPD with augmentation therapy.

Non AATD-related COPD patients had the highest AAT concentrations (149.3 mg/dL (SD: 23.8), p < 0.001) (Fig 2) (Table 2) as well as the highest absolute EIA (1.88 mg/mL (SD: 0.4) p < 0.001) compared to the AATD-related COPD group. On the other hand, and as expected, non-augmented Pi*ZZ patients had the lowest AAT concentrations (24 mg/dL (SD: 5.7) p < 0.001) and the lowest EIA (0.24 mg/ml (SD: 0.01) p < 0.001). Pi*ZZ patients on augmentation therapy showed values not significantly different from those observed in the control group (p < 0.05).

**Table 2:** Comparison of alpha1 antitrypsin (AAT) serum levels, elastase inhibitory activity (EIA) and the EIA/AAT ratio among groups.

Variables	Total (N = 86)	Control (N = 30)	COPD (N = 36)	Pi*ZZ not on augmentation (N = 9)	Pi*ZZ on augmentation (N = 11)	p value
AAT levels (mg/dL) Mean (SD) Median (IQR)	124.4 (46.2) 134 (111; 154)	128.6 (18.3) 124 (116; 141)	149.3 (23.8) 147 (130; 161)	24.0 (5.7) 22.6 (21; 23.5)	111.7 (60.9) 70.8 (62.6; 181)	<0.001^a^^bdf^
Absolut EIA (mg/mL) Mean (SD) Median (IQR)	1.46 (0.6) 1.49 (1.15; 1.82)	1.36 (0.24) 1.34 (1.18; 1.52)	1.88 (0.4) 1.8 (1.63; 1.98)	0.24 (0.01) 0.24 (0.24; 0.24)	1.3 (0.8) 0.82 (0.66; 2.23)	<0.001^a^^bdf^
EIA/total AAT ratio Mean (SD) Median (IQR)	1.16 (0.3) 1.11 (1.05; 1.22)	1.06 (0.15) 1.06 (0.97; 1.1)	1.27 (0.3) 1.2 (1.13; 1.26)	1.04 (0.2) 1.06 (1.02; 1.14)	1.14 (0.5) 1.06 (1.02; 1.15)	<0.001^a^^de^

Footnote Values are mean (standard deviation). COPD: chronic obstructive pulmonary disease; AAT: alpha1 antitrypsin; EIA: elastase inhibitory activity; SD: Standard Deviation. Results are expressed by means and standard deviation. P-values obtained from the Kruskal-Wallis test.

^a^P < 0.05 for comparison between Control and Non AATD-related COPD. ^b^ P < 0.05 for comparison between Control and Pi*ZZ not on augmentation therapy. ^c^ P < 0.05 for comparison between Control and Pi*ZZ on augmentation therapy. ^d^ P < 0.05 for comparison between Non AATD-related COPD and Pi*ZZ not on augmentation therapy. ^e^ P < 0.05 for comparison between Non AATD-related COPD and Pi*ZZ on augmentation therapy. ^f^ P < 0.05 for comparison between Pi*ZZ not on augmentation therapy and Pi*ZZ on augmentation therapy.

The EIA/AAT ratio was higher in non AATD-related COPD patients and in augmented Pi*ZZ patients (1.27 (SD: 0.3) and 1.14 (SD: 0.5) respectively) and lower in controls and non-augmented Pi*ZZ individuals (1.06 (SD: 0.15) and 1.04 (SD: 0.2), respectively) (p < 0.001) (Table 2 and Fig 2).

### Characteristics of non AATD-related COPD patients according to the EIA/AAT ratio

We divided the group of non AATD-related COPD according to the median value of the EIA/AAT ratio (1.3 mg/mL). COPD patients with a higher EIA/AAT ratio were significantly older (74.6 (SD: 7.1) vs. 68.2 years (SD: 12); p = 0.048), with higher comorbidity burden (CCI: 5.75 (SD: 1.5) vs. 4.8 (SD: 1.9), p = 0.040), lower AAT levels (mg/dL) (144.7 (SD: 27) vs. 150.1 (SD: 19), p = 0.001) and higher absolute EIA (mg/mL) (2 (SD: 0.4) vs. 1.7 (SD: 0.2), p = 0.001), with a trend towards higher disease severity (BODEx index: 3.6 (SD: 2.0) vs. 2.8 (SD: 1.9), p = 0.1). The distribution of the Pi*MM and Pi*MS genotypes was not significantly different between the two groups (Table 3).

**Table 3:** Sociodemographic, clinical variables and biomarkers in non-AATD related COPD patients according to levels of elastase inhibitory activity.

Variables	COPD with EIA < 1.3 mg/mL (N = 18)	COPD with EIA ≥ 1.3 mg/mL (N = 18)	p value
Age, years	68.2 (12.0)	74.6 (7.1)	**0.048**
Sex, male (%)	14 (77.8)	15 (78.9)	>0.999
Former smokers (%) Active smokers (%)	17 (94.4) 1 (5.6)	16 (88.9) 2 (11.1)	0.546
Smoking, pack-years	42.4 (22.7)	45.8 (16.8)	0.528
Charlson comorbidity index	4.80 (1.9)	5.75 (1.5)	**0.040**
FEV1 (%)	55.4 (22.3)	51.9 (19.5)	0.646
Patients with exacerbations in the previous year (%)	5 (27.8)	3 (16.7)	0.423
BMI (kg/m2)	28.6 (5.8)	27.7 (4.3)	0.862
CAT score	13.5 (5.6)	13.6 (6)	0.725
MRC dyspnoea score	2 (1.1)	2.5 (1)	0.250
BODEx index	2.80 (2)	3.55 (2)	0.100
Fibrinogen (mg/dL)	440.3 (48.5)	423.5 (85.2)	0.352
Haemoglobin (g/dL)	14.7 (2)	13.3 (2.8)	0.132
Leukocytes (x10^9/L)	7.85 (2.1)	7.81 (2.4)	0.987
C-reactive protein (mg/L)	1.59 (2.1)	0.60 (0.4)	0.350
AAT levels (mg/dL)	150.1 (19)	144.7 (27)	**0.001**
Absolut EIA (mg/mL)	1.72 (0.2)	2.01 (0.4)	**0.001**
EIA/total AAT	1.13 (0.1)	1.43 (0.4)	**0.038**

Footnote Values are mean (standard deviation) unless otherwise specified. BODEx: Body mass index, airflow Obstruction, Dyspnoea and Exacerbations; FEV1: Forced expiratory volume in 1 second; BMI: Body Mass Index; CAT: COPD Assessment Test; MRC: Modified Medical Research Council; AAT: alpha1 antitrypsin EIA: elastase inhibitory activity.^1^ p-values obtained from the Kruskal-Wallis test. ^2^ P-values obtained from the Chi-square test or the Fisher’s exact test. Significant differences in bold.

## Discussion

The results of our study reveal significant insights into the relationship between AAT levels, EIA, and the clinical characteristics of patients with COPD and severe AATD. The results demonstrated a strong correlation between AAT levels and EIA across all patient and control groups suggesting that the assay technique developed is robust and accurate for assessing EIA in diverse populations. Moreover, the positive association between higher AAT levels and increased EIA underscores the relevance of AAT as the main antiprotease and a key regulator of inflammatory processes in lung diseases. However, other antiproteases, such as elafin or secretory leukocyte protease inhibitor (SLPI), have also been described and may play a role in cases of deficient protection from AAT, either due to a genetic deficiency of AAT or an excess of demand due to a chronic inflammatory process [8]. It is known that AATD leads to a protease/antiprotease imbalance [27], as there is excessive NE that is not inhibited, overacting with its protease function, and destroying lung tissue contributing to the development of pulmonary emphysema.

Significant differences in absolute EIA between groups were observed in our study, with the highest levels found in non AATD-related COPD patients and the lowest in non-augmented AATD-related COPD individuals. These differences mimic those observed in serum AAT levels and underscore both: 1) the strong correlation between AAT levels and EIA, and 2) the role of AAT as the main source of EIA in human plasma.

Significant differences in the EIA/AAT ratio were also observed among groups. Specifically, non AATD-related COPD and augmented AATD-related COPD patients exhibited the highest ratios. These groups of patients are characterised by having more severe respiratory impairment and a heightened inflammatory state [28]. This elevated EIA/AAT ratio suggests that mechanisms other than increased AAT levels may contribute to the high EIA observed during inflammatory states associated with lung tissue damage. In fact, some signalling pathways can be altered in patients with severe pulmonary disease; specifically, higher expression of genes involved in inflammatory response-related signal transduction pathways, such as mitogen-activated protein kinase (MAPK), has been found in severe COPD patients [29]. These alterations may modify the expression of proteins involved in the protease/anti-protease balance. Additionally, in the context of chronic inflammation, the oxidative stress may play an important role in the decreased lung function by releasing reactive oxygen species (ROS) and the activation of transcription factors that can induce an overproduction of inflammatory cytokines and other proteins, creating a feedback loop of inflammation and oxidative stress that can modulate protease-antiprotease balance.

Patients with non-AATD related COPD had significantly higher serum concentrations of AAT compared to healthy individuals. This suggests that COPD patients may respond to a proinflammatory environment by increasing their AAT levels and consequently increasing their EIA. However, the increased EIA/AAT ratio observed in this population implies that antiproteases other than AAT may also play a role in this compensatory mechanism [6]. SLPI and elafin are members of the whey acidic four-disulfide-core proteins, a family of multifunctional host defence proteins that are expressed in response to various stimuli, mainly in macrophages and neutrophils [30]. Multiple functions are associated with these proteins, including anti-bacterial, anti-viral and anti-inflammatory properties, but they also possess the capacity to execute anti-protease activity against NE and other proteases [9]. Although AAT is the major inhibitor of NE, these other anti-proteases may significantly increase EIA in some specific conditions. Regarding augmented Pi*ZZ patients, the higher EIA can be attributed in part to the exogenous AAT from the augmentation therapy, but since they also have COPD, the increased EIA/AAT ratio may also be associated with the presence of other antiproteases, as in non-AATD related COPD.

Interestingly, despite the expected reduction in EIA observed in patients with non-augmented AATD-related COPD due to the extremely low concentrations of plasma Z AAT, the EIA/AAT ratio in these patients was not significantly reduced. This contrasts with previous studies that demonstrated a lower EIA of the Z AAT protein compared with the wild type M [9]. Again, the role of other antiproteases may account for the preserved EIA/AAT ratio despite the reduced enzymatic activity of the Z protein, since most of these individuals were also affected by significant respiratory disease and, therefore, the same inflammatory stimuli as in non-deficient COPD may apply. It is important to consider that our measurements correspond to circulating AAT, which is not affected by possible functional inhibition by the local inflammatory environment in the lung. Similar studies in the pulmonary environment, such as in bronchoalveolar lavage fluid, could provide more insights into the possible functional alteration of normal (M) and deficient (Z) AAT in patients with COPD [31].

According to our results, we can hypothesize that a compensatory mechanism involving other antiprotease strategies may be especially significant in individuals with more severe manifestations of pulmonary disease, such as advanced emphysema, [27], but may also exist to a lesser extent in early stages of pulmonary disease. Thus, our study underscores the multifaceted nature of antiprotease activity in modulating EIA under various pathological conditions.

Within the non AATD-related COPD group, patients were stratified based on the median value of the EIA/AAT ratio. The subgroup with a higher EIA/AAT ratio corresponded to patients with lower AAT levels, more severe lung disease and a higher comorbidity burden. The higher EIA/AAT ratio observed in this group, and especially in the two patients with severe comorbidities, suggests that the additional mechanisms involving other antiproteases may be stimulated by the same increased inflammatory state associated with the development of comorbidities in COPD [32]. These results reinforce the concept of the EIA/AAT ratio as a useful marker of disease severity and prognosis in COPD patients.

Some smokers with normal serum AAT levels may develop COPD of a similar degree of severity compared to patients with AATD [33]. In these cases, the determination of EIA may provide relevant additional information to the isolated determination of serum AAT levels. The lack of enough antiprotease protection, despite having normal serum AAT values, may make these subjects future candidates for treatment with the oral inhibitors of neutrophil elastase currently under development [34].

Our study has some limitations: 1) it was a cross-sectional study. Future longitudinal studies are needed to allow a more comprehensive analysis of the dynamic impact of EIA as a biomarker in the prognosis of lung impairment. 2) Since AATD is a rare disease, our group of patients with the Pi*ZZ genotype was limited. However, the results obtained in this population with and without augmentation therapy were very consistent, although larger studies would be required to confirm these findings. 3) Assessing EIA at the pulmonary level may reveal different results and more differences between groups. Measuring EIA in sputum samples could provide a more accurate estimate of the protease-antiprotease balance within the lungs. This could clarify whether serum EIA levels truly correspond to local pulmonary dynamics or if there are distinct regulatory mechanisms that play a role in lung tissue.

In conclusion, the present study underscores the complex interplay between AAT levels, EIA and disease severity in respiratory conditions such as COPD associated or not with AATD. The observed variations in EIA across patient groups highlight the potential of EIA as a biomarker for assessing disease progression and therapeutic needs to restore the antiprotease protection. EIA monitoring could provide valuable insights into patient-specific therapeutic needs, helping to perform a more personalized treatment for each patient. By assessing disease progression and therapy effectiveness, this biomarker could play a crucial role in optimizing treatment strategies, particularly in patients that are under augmentation therapy but they are not demonstrating a favourable clinical course. Moreover, the easy implementation and the reasonably cost-efficiency throughput of the technique used to measure EIA makes it an interesting additional diagnostic test in the assessment of severe COPD.

## Supporting information

S1 File Raw Data Elastase Plos One V2. Anonymized participant background information.
(XLSX)
